# Absolute quantitative lipidomics reveals lipids profiling in liver of mice with early-stage alcoholic liver disease

**DOI:** 10.1186/s12986-022-00679-z

**Published:** 2022-07-05

**Authors:** Fei Zhao, Jun Chen, Rui Guo, Jinyan Zhu, Weijia Gu, Songtao Li, Jiaomei Li

**Affiliations:** School of Public Health, Zhejiang Chinese Medical University, 488 Binwen Road, Hangzhou, 310053 China

**Keywords:** Alcoholic liver disease, Lipidomics, Alcohol intake, Triglyceride, Free fatty acids

## Abstract

**Background:**

Alcoholic liver disease (ALD) is one of the most prevalent chronic liver disease worldwide. Alcohol-induced alterations in hepatic lipids play an important role in ALD develpoment and progression. The present study aimed to thoroughly describe the changes of lipid profiling in liver of mice with early-stage alcoholic liver disease.

**Methods:**

C57BL/6J male mice aged 7-week were randomized into alcohol-fed (AF) group and pair-fed control group (PF) (n = 10 per group). The early stage of ALD was induced with Lieber-DeCarli liquid diet. The lipids profiling was analyzed by absolute quantitative lipidomics with UHPLC-QTRAP-MS/MS.

**Results:**

Alcohol intake significantly increased the levels of alanine aminotransferase (ALT) in plasma, and tumor necrosis factor-α (*TNF-α*), interleukin-6 (*IL-6*) and triacylglycerols (TAG) levels in liver. Lipidomis analyses showed that 41 TAGs were up-regulated and 8 TAGs were down-regulated in response to alcohol intake. The 8 decreased TAGs were with more double bond, longer carbon chain length and mostly contained docosahexaenoic acid (C22:6n-3) and eicosapentaenoic acid (C20:5n-3), compared with the up-regulated TAGs. Furthermore, the down-regulated TAG(56:9)_FA20:5 was inversely associated with ALT and *IL-6* levels. In addition, several altered lysophosphatidylcholines (LPC), lysophosphatidylethanolamines (LPE) and hexosylceramides (HCER) were all significantly decreased in response to alcohol consumption, especially HCer(18:1/22:0), with the top reduction among all the down-regulated lipids.

**Conclusions:**

These findings suggest that not only the up-regulated lipids, alcohol-induced reduction in some specific lipids might also contribute to the ALD development, especially TAG(56:9)_FA20:5 and HCer(18:1/22:0). Their physiological functions and effects on ALD development warrants further investigation.

**Supplementary Information:**

The online version contains supplementary material available at 10.1186/s12986-022-00679-z.

## Background

Alcoholic liver disease (ALD), commonly induced by long-term alcohol abuse, is one of the most prevalent liver diseases and the leading cause of mortality of liver-related disease globally, especially in developing countries [1]. ALD is an umbrella term for a group of liver diseases, ranging from early-stage steatosis, steatohepatitis, to late-stage fibrosis, cirrhosis and hepatocellular carcinoma [2]. Early stages of ALD are often overlooked by patients and are prone to progress to advanced liver disease, which has been an important cause of liver transplantation in recent years [3]. Abstinence is supposed to be the most effective way to reverse the development and progression of ALD. However, it is too hard to be achieved by most heavy drinkers. Clinically, to date, there is no Food and Drug Administration-approved medications to halt this progress. The increasing global burden of ALD highlights the urgent need for more understanding about this disease and its exact pathogenesis.

Generally, alcohol can be metabolized to acetaldehyde in liver firstly, and then metabolized to ethanoic acid in mitochondria. This process consumes a lot of nicotinamide adenine dinucleotide, which results in the inhibition of β-oxidation of fatty acids, and ultimately leads to hepatic steatosis [4]. Hepatic steatosis is a direct consequence of alcohol metabolism and is considered as a benign condition due to its reversible character [5]. However, with the continuous lipids accumulation in liver, the hepatocytes’ ability of resisting risk weakens gradually, increasing the probability of liver injury. Damage-associated molecular patterns activated by the injured hepatocytes further promote the release of inflammatory factors, aggravating the progression of ALD [6]. Therefore, inhibiting hepatic lipid accumulation is important to break the vicious circle and is effective to reduce liver injury and inflammatory response, finally improving ALD.

Evidences from animal experiments have shown the complex interaction between alcohol and hepatic lipid metabolism [7]. It has been proposed that alcohol has distinct effects on different types of fatty acids, according to the carbon length and degree of unsaturation [8]. Besides, alcohol may influence the metabolisms of many lipid classes, such as lysophosphatidylcholines (LPC), lysophosphatidylethanolamines (LPE) and ceramides [9]. Furthermore, a high-fat diet rich in n-6 polyunsaturated fatty acids significantly aggravated alcohol-induced liver injury and steatosis, while the n-3 polyunsaturated fatty acids showed the opposite effect [10, 11]. Even though it has been confirmed that alcohol-induced lipid changes have an important role in the progress of ALD, studies targeting at the description of lipid changes in response to alcohol intake are still limited.

Recent advances in the science of lipidomics has allowed for the global description and identification of lipid species at systems and molecular level [12]. A previous study has found that lipidomics analysis can identify various stages of non-alcoholic fatty liver disease [13]. However, using lipidomics techniques to assess the lipid profiles in the early stages of ALD, especially hepatic steatosis, is less documented.

This study aims to thoroughly describe the lipids changes in liver tissue in response to chronic alcohol consumption using method of absolute quantitative targeted lipidomics. In addition, the association between significantly changed lipids and indicators of liver injury, steatosis, lipid peroxidation and inflammation were analyzed to assess the role of the specific lipids in the pathogenesis of ALD.

## Material and method

### Mice and diet

A total of 20 C57BL/6J male mice aged 7 weeks were purchased from B&K Experimental Animal Corporation Ltd (Shanghai, China). All of them were housed in Zhejiang Chinese Medical University Animal Facility with specific pathogen-free conditions (five mice/cage; 12 h light–dark cycle; 22 ± 1 °C; 60%-65% humidity). They were cared for in according to the Guide for the Care and Use of Laboratory Animals.

After a week of habituation, mice were randomized into alcohol-fed group (AF) and pair-fed control group (PF). Mice in the control group took the control Lieber-DeCarli liquid diet, with 47% energy from carbohydrate, 18% energy from protein and 35% energy from fat. In the alcohol-fed group, equicaloric maltose dextrin was substituted with 95% ethanol to maintain the isoenergy intake between two groups. And energy percentage of alcohol in total energy was gradually increased from 0% in the initial 1–3th days, to 5.5% in the 4–5th days, to 11% in the 6–7th days, to 22% in the second week, to 27% of total energy in the third week, to 32% of total energy in the fourth week finally. It is a mature method in our lab to induce the mice model of alcoholic fatty liver disease. The detail composition of the diet was shown in Table 1. Dietary intake and body weight were recorded twice in each week.

**Table 1:** The detail composition of the diet

	Time	Calories from alcohol (%)	95% ethanol (mL)	Maltodextrin 10 (g)	Corn oil (g)	Vitamin mix (g)	Choline (g)	Feed powder (g)
PF	1–4 weeks	0	0	115.20	39.59	2.50	0.52	64.00
AF	1–3 days	0	0	115.20	39.59	2.50	0.52	64.00
	4–5 days	5.5	10.47	101.66				
	6–7 days	11.0	20.94	88.11				
	2 week	22.0	41.88	61.02				
	3 week	27.0	51.40	48.70				
	4 week	32.0	60.91	36.39				

*PF* pair-fed control group, *AF* alcohol-fed group

At the end of 4 weeks’ intervention, all mice were sacrificed under anesthesia after fasting for 12 h. Blood and liver tissues were collected for further analysis, including the following biochemical analysis, histological assessment, qRT-PCR and lipidomics analysis.

### Biochemical analysis

Blood sample was centrifuged with 2000 rpm for 15 min at 4 °C, and plasma was separated and then kept at − 80 °C until use. The concentrations of aspartate aminotransferase (AST), alanine aminotransferase (ALT), total cholesterol (TC) and triacylglycerol (TAG) in plasma were determined with an automatic analyzer (Model XE-2100, Sysmex Kobe, Japan).

Liver tissue samples were homogenized in methanol and centrifuged for 15 min at 3000 rpm. The supernatant was separated for detecting hepatic TC and TG concentrations with enzymatic colorimetric methods using commercially available kits (Nanjing Jiancheng Bioengineering Institute, Jiangsu, China). Another liver sample was homogenized in saline and then centrifuged. The supernatants were separated for investigating the concentrations of superoxide dismutase (SOD) and malondialdehyde (MDA) with commercial kits (Lianke Biotechnology CO., Ltd., Zhejiang, China).

### Histological assessment

Liver specimens were fixed in 10% formalin, then dehydrated and embedded in paraffin. The frozen paraffin blocks were sectioned and subsequently stained with Haematoxylin and Eosin (H&E) for histological examination. In addition, another liver samples were embedded in Tissue-Tek OCT, then sectioned in 4–5 μm thick sections, and stained with Oil Red O (Sigma-Aldrich, St. Louis, MO, USA) for the evaluation of fat deposition. Finally, they were viewed under a light microscope (100× and 200×) and analyzed via Image J software.

### Quantitative real-time reverse transcription polymerase chain reaction

Total RNA was purified from liver tissue using Trizol reagent (Thermo, Waltham, MA, USA), according to the manufacturers’ instructions. Then cDNA was synthesized with Prime Script RT Reagent kit (Takara Biotechnology Co., Ltd, Dalian). Quantitative real-time PCR was performed in 7500 Fast Real-Time PCR System (Applied Biosystems, Waltham, MA, USA) using SYBR Premix Ex Taq TM (Tli RNaseH Plus) kit (Takara Biotechnology Co., Ltd, Dalian), as described by the manufacturer. The sequences of primers for real-time PCR of *IL-6* were 5′-CCGGAGAGGAGACTTCACAG-3′ (forward) and 5′-CAGAATTGCCATTGCACAAC-3′ (reverse); for real-time PCR of *TNF-α* were 5′-CCCTCACACTCACAAACCAC-3′ (forward) and 5′-ACAAGGTACAACCCATCGGC-3′ (reverse). The levels of gene expression in control group were set to 1.0, and results from the other group were shown as relative expression ratios to the control group.

### Lipidomics analysis

Liver samples were weighed and then cut into small sections (n = 6 per group). Sections from the same place of each liver sample were prepared for lipidomics analysis with ExionLC UHPLC system coupled to QTrap 6500 + mass spectrometer (SCIEX, USA). A mixture of 10 mg liver sample and 400 μL water was homogenized for 60 s, and then ground with a mixer mill at 45 Hz for 4 min, followed by ultrasonic treatment for 5 min in ice-water bath. Then a mixture of 10 μL homogenate, 190 μL water and 480 μL extract solution (methyl tert-butyl ether: methanol = 5:1) containing internal standard was vortexed vigorously and centrifuged at 3,000 rpm for 15 min at 4 °C. After that, 250 μL of supernatant was transferred into a fresh tube and dried in vacuum at 4 °C. The dried samples were dissolved in 100 μL of solution (DCM: methanol: water = 60: 30: 4.5), and then centrifuged with 12,000 rpm for 15 min at 4 °C. The supernatant was transferred into a fresh glass vial for further analysis. In addition, the quality control (QC) sample was prepared by pooling 10 μL of supernatants from each sample together.

2 μL of the sample solution was injected into the liquid chromatographic column (Acquity HSS T3 Column, 1.8 μm, 2.1 mm × 100 mm). The mobile phase A consisted of 60% acetonitrile, 40% water and 10 mmol/L ammonium formate. The mobile phase B consisted of 90% isopropanol, 10% acetonitrile and 10 mmol/L ammonium formate. The elution program was as follows: 20% B in 0–1 min, 20–60% B in 1–4 min, 60–98% B in 4–15 min, 98% B in 15–16 min, 20% B in 16.1–18 min. The flow rate was 0.3 mL/min, and temperature of column and auto-sampler were set at 40 °C and 6 °C, respectively. During sequence analysis, QC sample was injected after every six samples to detect the reproducibility of sample and the stability of analytical platform.

Sciex QTrap 6500 + MS was conducted in both positive-ion and negative-ion modes. Primary ion source parameters were as follows: Ion-spray voltage, +5500/−4500 V; Curtain gas, 40 psi; Temperature, 350 °C; Ion source gas, 1: 50 psi; Ion source gas, 2: 50 psi.

### Data preprocessing

The raw data were imported into Skyline 20.1 to quantify the target compounds. The features would be excluded if they were present in less than 50% of the quality control samples, or if they showed a coefficient of variation of more than 20% in quality control samples. Features with a peak area less than 3000 in 80% of all samples were regarded as non-detected. The internal standards used in this method were listed in Additional file 1: Table S1. The absolute content of each lipid was calculated according to the peaks area and actual concentration of the internal standard in each lipid class.

### Statistical analysis

Biochemical data were presented as means ± standard deviations (SD). The statistical significance between two groups were tested with Student’s t-test. Difference of *P*-value less than 0.05 was considered statistically significant. The analysis was performed with SPSS 23.0 software.

For data of lipidomics metabolites, the dataset containing sample name, peak number and normalized peak area were imported to SIMCA-P version 16.0.2 for multivariate analysis. Firstly, values of features were log_10_-transformed and subjected to Unit Variance Scaling. Then principal component analysis (PCA), an unsupervised multivariate pattern recognition analysis, was conducted to visualize the clustering of the samples from same group and the QC samples. Secondly, a supervised multivariate orthogonal partial least squares discriminant analysis (OPLS-DA) was conducted to discriminate the separation of lipid profiling between two groups. The quality of the fitted model was assessed with R^2^Y and Q^2^Y parameters, which mean the goodness of fit and prediction, respectively. Then a 200 times permutation test was conducted to further check the robustness and predictive ability of the obtained OPLS-DA model. The value of variable influence on the projection (VIP) of the first principal component was obtained from OPLS-DA. The values summarize the contribution of each variable to the model. Only the metabolites with a VIP value higher than 1 and *P* < 0.05 (student’s t-test) were considered as significantly different. Furthermore, correlations between lipids and ALD indicators were investigated with the Pearson’s correlation test.

## Results

### Effect of alcohol intake on liver injury, hepatic steatosis, lipid peroxidation and inflammation level in mice

The food consumption, body and liver were weighted. The levels of food consumption and body weight were similar between alcohol-fed and pair-fed mice, whereas liver weight, and ratio of liver/body weight were significantly higher in alcohol-fed mice than in control mice (Table 2).

**Table 2:** Effect of alcohol intake on body/liver weights and indicators of liver injury, lipid peroxidation and hepatic steatosis (n = 10 per group)

	Pair-fed group	Alcohol-fed group	*P* value
Body weight (g)	23.10 ± 1.77	23.50 ± 1.22	0.32
Liver weight (g)	0.84 ± 0.09	1.09 ± 0.05	< 0.001
Liver/Body weight	0.04 ± 0.00	0.05 ± 0.00	< 0.001
Plasma AST (IU/L)	40.66 ± 7.48	39.07 ± 13.40	0.83
Plasma ALT (IU/L)	39.60 ± 10.88	67.18 ± 17.78	0.049
Plasma TC (μM)	925.0 ± 55.27	932.3 ± 51.95	0.84
Plasma TAG (μM)	484.8 ± 108.2	943.5 ± 12.51	< 0.001
Liver TC (μM/g)	5.19 ± 0.37	5.30 ± 0.24	0.56
Liver TAG (μM/g)	21.07 ± 6.05	29.68 ± 3.59	0.02
MDA (nmol/mg)	4.89 ± 0.78	14.66 ± 2.31	< 0.001
SOD (U/mg)	0.47 ± 0.11	0.32 ± 0.06	< 0.001

*AST* aspartate aminotransferase, *ALT* alanine aminotransferase, *TC* total cholesterol, *TAG* triacylglycerol, *MDA* malondialdehyde, *SOD* superoxide dismutase

Plasma AST and ALT, as key indicators of liver injury, were determined. The results showed that ALT level in plasma of alcohol-fed mice was significantly increased compared with control mice. Even though there was no significant difference in AST level between two groups, the data still robustly indicated the liver injury induced by alcohol intake.

**Fig. 1:**
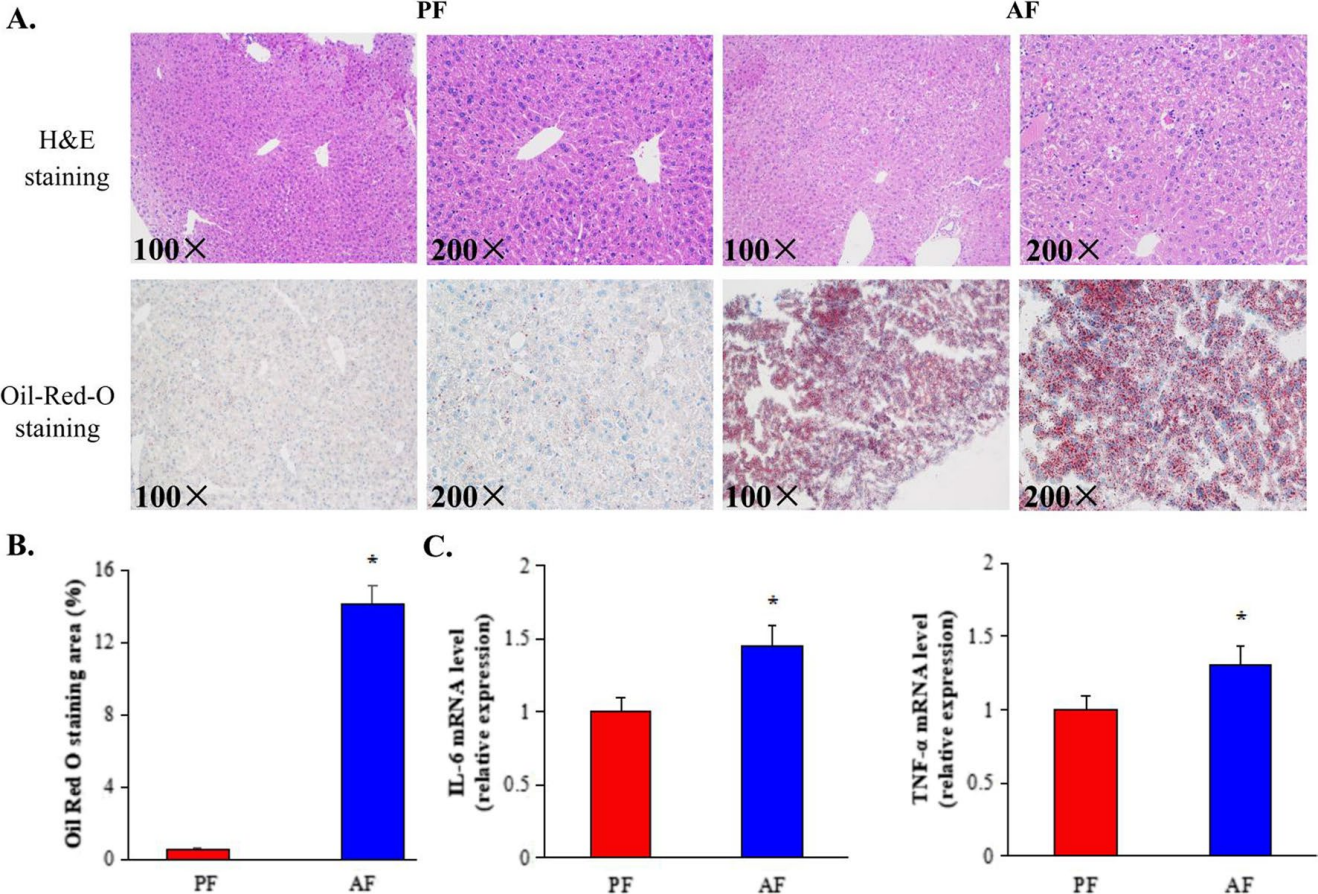
Effect of chronic alcohol intake hepatic steatosis and liver injury. **A** Representative images of hepatic hematoxylin and eosin (H&E) and of Oil Red O staining (100× and 200×). **B** Quantification of lipid accumulation based on Oil red O staining (200×). **C** Changes of inflammation in mice liver in response to alcohol intake (n = 10 per group). *PF* pair-fed control group, *AF* alcohol-fed group

Histological examinations of both hepatic H&E staining and Oil-Red-O staining showed aggravated hepatic steatosis and liver injury in response to alcohol exposure (Fig. 1A). TC levels in liver and plasma showed no difference between alcohol-fed and pair-fed mice, whereas TAG levels in liver and plasma were significantly increased in alcohol-fed mice compared with control group.

To investigate the effect of alcohol intake on lipid peroxidation, hepatic levels of SOD and MDA were determined. The results showed that SOD was significantly decreased in response to alcohol exposure. MDA was sharply increased in AF group compared with control mice (Table 2).

To investigate the effect of alcohol intake on inflammation in mice liver, levels of *TNF-α* and *IL-6* mRNAs were determined. The results showed that levels of both *TNF-α* and *IL-6* were significantly increased in alcohol-fed mice compared with control mice (Fig. 1B). Taken together, all these data demonstrate that alcohol intake significantly aggravated liver injury, hepatic steatosis and inflammation in mice liver.

### Alcohol-induced changes of lipids profiling in mice liver

**Fig. 2:**
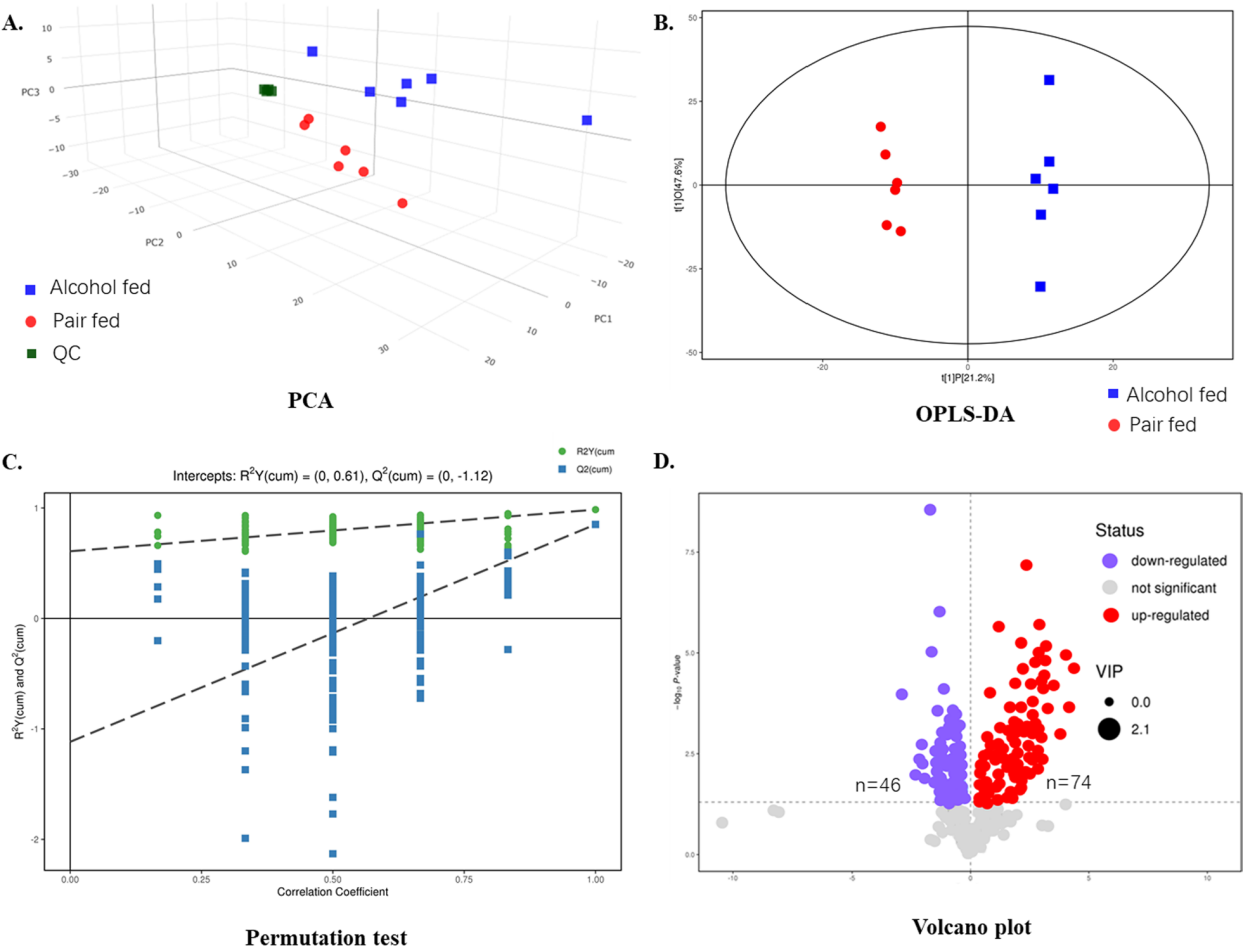
Alcohol-induced changes of lipids profiling in mice liver (n = 6 per group). **A** Score plot of principle component analysis (PCA) in alcohol-fed, pair-fed and quality control groups. **B** Score plot of orthogonal partial least squares discriminant analysis (OPLS-DA) in alcohol-fed and pair-fed control groups. **C** Permutation tests for OPLS-DA model. **D** Volcano plot showing changes of 120 lipids in mice liver after alcohol intervention. The up-regulated lipids were colored with red, the down-regulated lipids were colored with blue, and the gray plots represent lipids not significantly changed

After preprocessing of raw data from detection, a total of 583 features were subjected to multivariate analysis. As shown in Fig. 2A, samples in each group were clustered in the PCA model. The three QC samples were closely clustered, suggesting a robust stability and reproducibility of instrumental analysis, and indicating a good quality of data acquisition.

After PCA, OPLS-DA was then conducted. The OPLS-DA score plot displayed a clear separation between alcohol-fed group and control group, indicating that lipids profiling was significantly changed by alcohol intake (Fig. 2B). R^2^Y and Q^2^Y values were 0.993 and 0.919, demonstrating the perfect predictive ability and stability of the obtained OPLS-DA model. Then 200 times permutation test were conducted to further confirm the model’s validity (Fig. 2C).

The lipids items with VIP value > 1 in OPLS-DA model were then compared between two groups with *t*-test, and 120 kinds of lipids were significantly changed in response to alcohol intake. Among them, 74 lipids were up-regulated and 46 lipids were down-regulated in alcohol-fed group compared with pair-fed control group (Fig. 2D). The up-regulated lipids consist of 41 TAGs, 10 diacylglycerols, 8 phosphatidylethanolamines, 5 ceramides, 2 dihydroceramides, 3 phosphatidylcholines, 2 sphingomyelins, 2 free fatty acids and 1 cholesterol esters. The down-regulated lipids consist of 8 TAGs, 9 phosphatidylethanolamines, 5 phosphatidylcholines, 6 hexosylceramides, 5 cholesterol esters, 3 lysophosphatidylcholines, 3 lysophosphatidylethanolamines, 3 sphingomyelins, 2 diacylglycerols and 2 ceramides. The compounds with the top VIP values for down-regulated lipid and up-regulated lipid are HCer(18:1/22:0) and FFA(20:5), respectively. Except the changed TAGs, all the down-regulated lipids and up-regulated lipids as well as their detail information are listed in Tables 3 and 4, respectively.

**Table 3:** The down-regulated lipids in mice liver in response to alcohol intake

No.	Name	Type	VIP value	Log_10_ (Fold change)	*P*-value
1	PE(16:0/22:4)	PE	2.079	− 1.379	< 0.001
2	PE(16:0/20:2)	PE	1.998	− 1.118	< 0.001
3	PE(18:1/20:2)	PE	1.823	− 0.747	0.008
4	PE(16:0/20:1)	PE	1.814	− 0.688	0.006
5	PE(16:0/16:1)	PE	1.266	− 0.654	0.037
6	PE(18:2/16:1)	PE	1.441	− 0.647	0.015
7	PE(18:1/20:1)	PE	1.644	− 0.498	0.006
8	PE(18:0/22:4)	PE	1.326	− 0.377	0.030
9	PE(18:1/20:4)	PE	1.544	− 0.368	0.011
10	DAG(18:1/20:1)	DAG	1.481	− 0.615	0.009
11	DAG(16:0/16:0)	DAG	1.424	− 0.289	0.021
12	PC(16:0/14:0)	PC	1.340	− 0.871	0.032
13	PC(16:1/18:1)	PC	1.875	− 0.792	< 0.001
14	PC(16:0/16:1)	PC	1.778	− 0.671	0.003
15	PC(16:1/18:2)	PC	1.859	− 0.618	< 0.001
16	PC(14:0/18:2)	PC	1.640	− 0.559	0.006
17	CE(16:1)	CE	1.782	− 1.336	0.011
18	CE(22:1)	CE	1.739	− 0.938	0.001
19	CE(22:2)	CE	1.773	− 0.779	0.002
20	CE(20:1)	CE	1.885	− 0.736	< 0.001
21	CE(20:2)	CE	1.415	− 0.573	0.036
22	Cer(18:1/22:1)	CER	1.895	− 0.892	< 0.001
23	Cer(18:1/14:0)	CER	1.350	− 0.332	0.024
24	SM(22:0)	SM	1.836	− 0.423	< 0.001
25	SM(22:1)	SM	1.785	− 0.419	0.006
26	SM(24:1)	SM	1.369	− 0.299	0.027
27	HCer(18:1/22:0)	HCER	2.133	− 1.722	< 0.001
28	HCer(18:0/22:0)	HCER	2.011	− 1.649	< 0.001
29	HCer(18:1/20:0)	HCER	2.045	− 1.340	< 0.001
30	HCer(18:0/24:0)	HCER	1.378	− 0.423	0.042
31	HCer(18:1/24:0)	HCER	1.587	− 0.422	0.006
32	HCer(18:1/26:0)	HCER	1.354	− 0.361	0.021
33	LPC(20:1)	LPC	1.842	− 0.889	< 0.001
34	LPC(16:0)	LPC	1.525	− 0.403	0.009
35	LPC(18:1)	LPC	1.259	− 0.272	0.043
36	LPE(16:1)	LPE	1.634	− 1.207	< 0.001
37	LPE(20:1)	LPE	1.814	− 0.752	< 0.001
38	LPE(20:2)	LPE	1.275	− 0.516	0.036

*PE* phosphatidylethanolamines, *DAG* diacylglycerols, *CER* ceramides, *CE* cholesterol esters, *PC* phosphatidylcholines, *SM* sphingomyelins, *HCER* hexosylceramides, *LPC* lysophosphatidylcholines, *LPE* lysophosphatidylethanolamines

**Table 4:** The up-regulated lipids in mice liver in response to alcohol intake

No.	Name	Type	VIP	Log_10_(Fold change)	*P*-value
1	PE(18:0/20:3)	PE	1.565	0.379	0.006
2	PE(18:1/18:2)	PE	1.644	0.462	0.009
3	PE(P-18:0/18:2)	PE	1.616	0.538	0.004
4	PE(18:0/18:1)	PE	1.818	0.753	0.001
5	PE(18:0/22:6)	PE	1.932	0.811	< 0.001
6	PE(18:1/20:5)	PE	1.399	0.916	0.002
7	PE(18:0/18:2)	PE	2.020	1.157	0.002
8	PE(18:0/20:5)	PE	1.975	1.201	< 0.001
9	DAG(18:0/18:1)	DAG	1.599	0.599	0.024
10	DAG(18:2/18:3)	DAG	1.476	0.820	0.029
11	DAG(18:1/20:5)	DAG	1.595	1.064	0.048
12	DAG(18:0/18:3)	DAG	1.920	1.083	0.004
13	DAG(18:1/22:6)	DAG	1.811	1.165	0.018
14	DAG(18:0/18:2)	DAG	1.974	1.167	0.003
15	DAG(18:0/22:6)	DAG	2.001	1.226	< 0.001
16	DAG(16:0/20:5)	DAG	1.808	1.393	0.002
17	DAG(18:2/22:6)	DAG	2.014	2.008	0.009
18	DAG(18:2/20:5)	DAG	1.908	2.064	0.026
19	PC(18:1/18:2)	PC	1.552	0.422	0.008
20	PC(18:2/18:2)	PC	1.647	0.458	0.006
21	PC(18:1/18:1)	PC	1.463	0.594	0.007
22	CE(20:5)	CE	1.930	1.632	< 0.001
23	Cer(18:1/26:0)	CER	1.212	0.311	0.048
24	Cer(18:1/16:0)	CER	1.527	0.507	0.031
25	Cer(18:1/26:1)	CER	1.615	0.638	0.003
26	Cer(18:1/18:0)	CER	1.715	0.654	0.016
27	Cer(18:1/24:0)	CER	1.762	0.835	0.003
28	Cer(18:0/18:0)	DCER	1.176	0.770	0.047
29	Cer(18:0/24:0)	DCER	1.597	0.975	0.045
30	SM(14:0)	SM	1.399	0.313	0.017
31	SM(24:0)	SM	1.552	0.357	0.009
32	FFA(22:6)	FFA	1.807	1.075	0.021
33	FFA(20:5)	FFA	2.044	1.776	0.001

*PE* phosphatidylethanolamines, *DAG* diacylglycerols, *PC* phosphatidylcholines, *CE* cholesterol esters, *CER* ceramides, *DCER* dihydroceramides, *SM* sphingomyelins, *FFA* free fatty acids

**Fig. 3:**
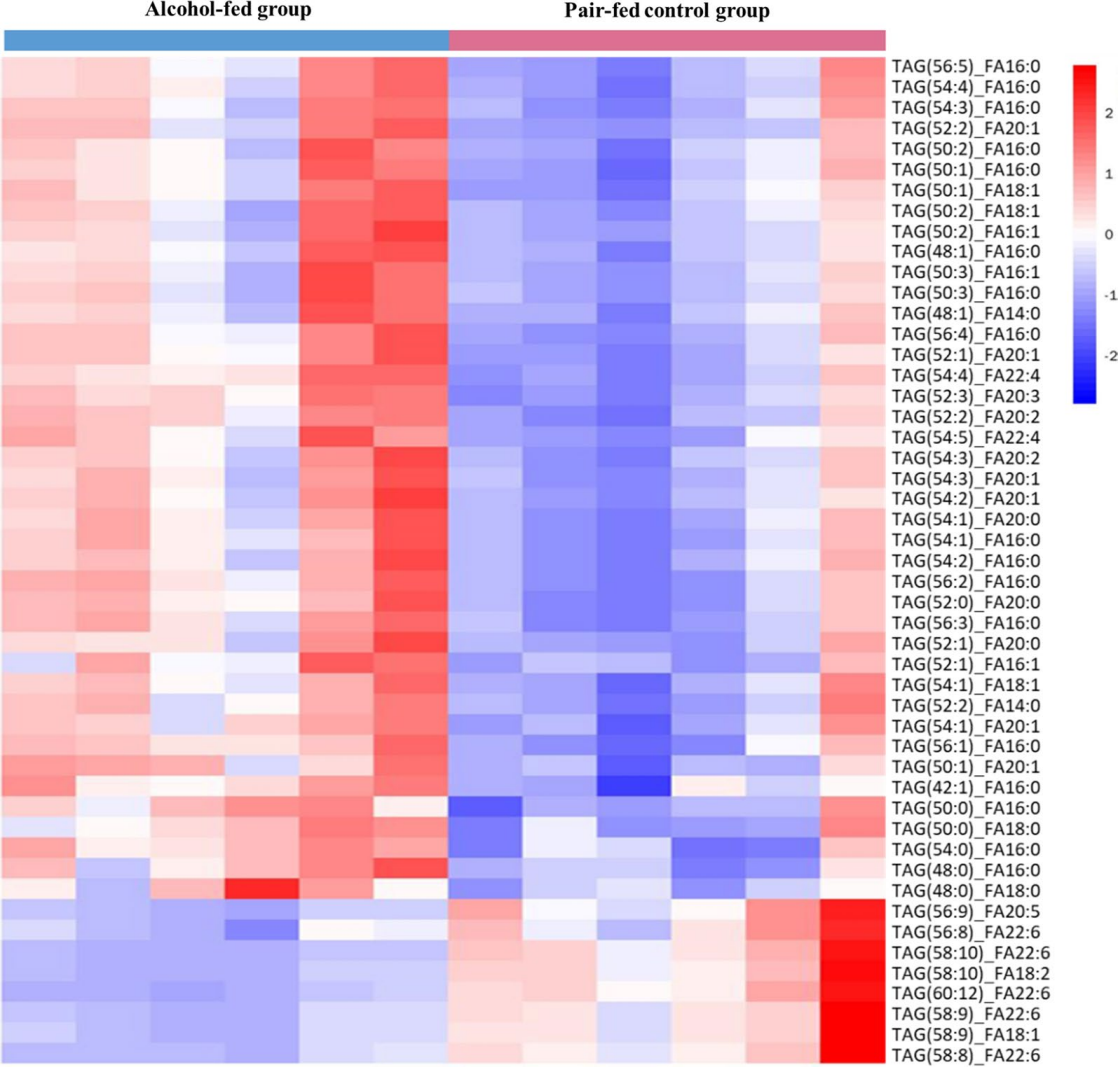
Heatmap of the 49 significantly differential triacylglycerols (TAGs) showed apparent clustering according to alcohol-fed and pair-fed groups (n = 6 per group). Red shows higher expression and blue shows lower expression

**Fig. 4:**
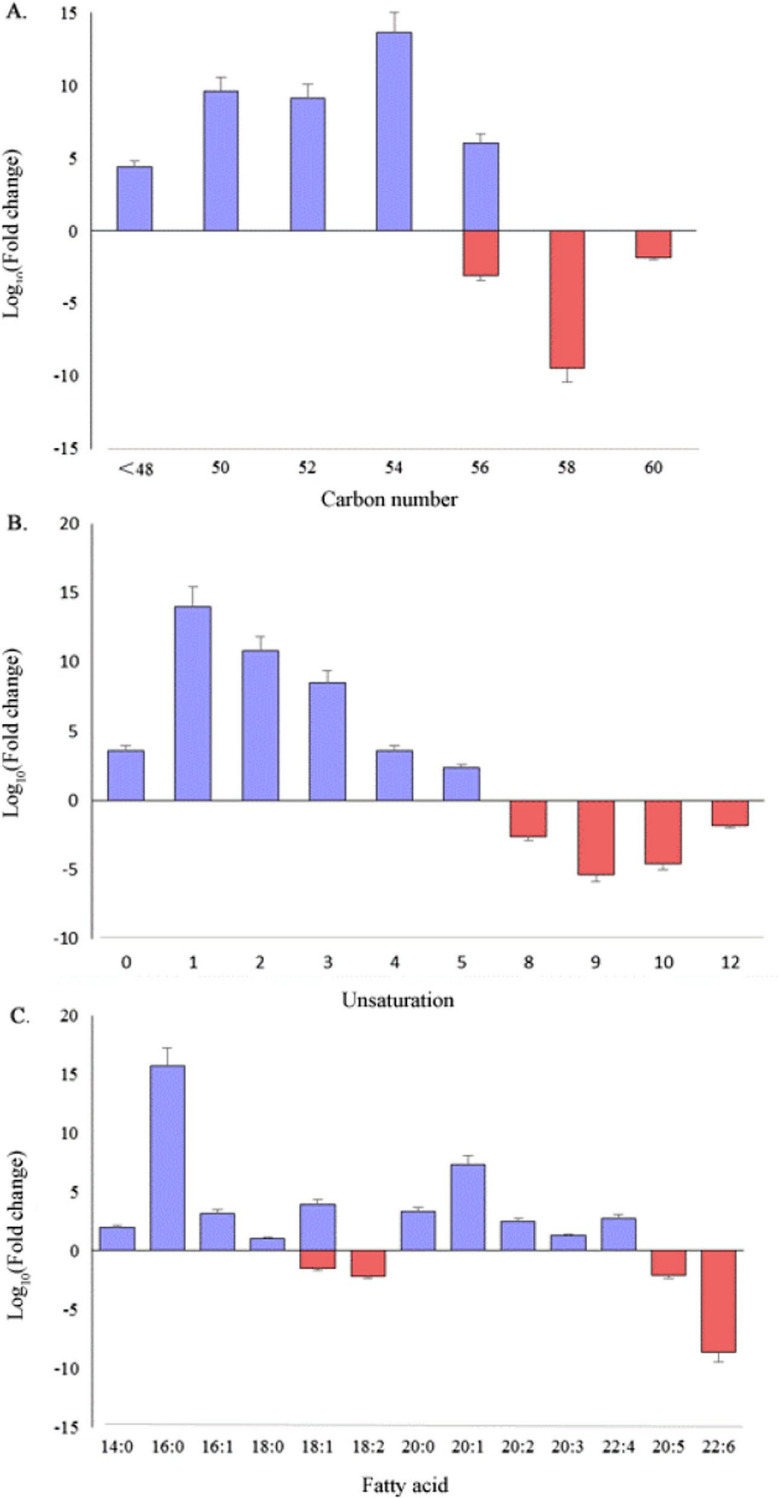
Characteristics of the 41 up-regulated TAGs and the 8 down-regulated TAGs in response to alcohol intake, according to their total carbon number (**A**), unsaturation (**B**) and contained fatty acid (**C**)

As TAG accounts for the largest proportion of the altered lipids, we then investigated this lipids species in detail. Heatmap of the 49 significantly differential TAGs showed apparent clustering between samples from alcohol-fed mice and pair-fed control mice (Fig. 3). To further characterize the 41 up-regulated TAGs and the 8 down-regulated TAGs in response to alcohol intake, we analyzed them in terms of unsaturation, carbon number and fatty acid composition. When comparing TAGs based on total carbon number, the results showed that alcohol intake mainly up-regulated TAGs with 54, 52 or 50 carbons, whereas down-regulated TAGs with 58 carbons (Fig. 4A). When comparing TAGs based on unsaturation, the results showed that alcohol intake mainly up-regulated the TAGs that had 1, 2 or 3 sites of unsaturation, while down-regulated the TAGs that had 9 or 10 sites of unsaturation (Fig. 4B). When compared from the view of fatty acids types, the results showed that alcohol intake mainly up-regulated TAGs containing palmitic acid (C16:0), eicosenoic acid (C20:1), palmitoleic acid (C16:1) and oleic acid (C18:1), whereas down-regulated TAGs containing docosahexaenoic acid (C22:6), eicosapentaenoic acid (C20:5) and linoleic acid (C18:2) (Fig. 4C).

### The correlation between the indicators of liver injury/steatosis/lipid peroxidation/inflammation and the top 25 changed triacylglycerols

**Fig. 5:**
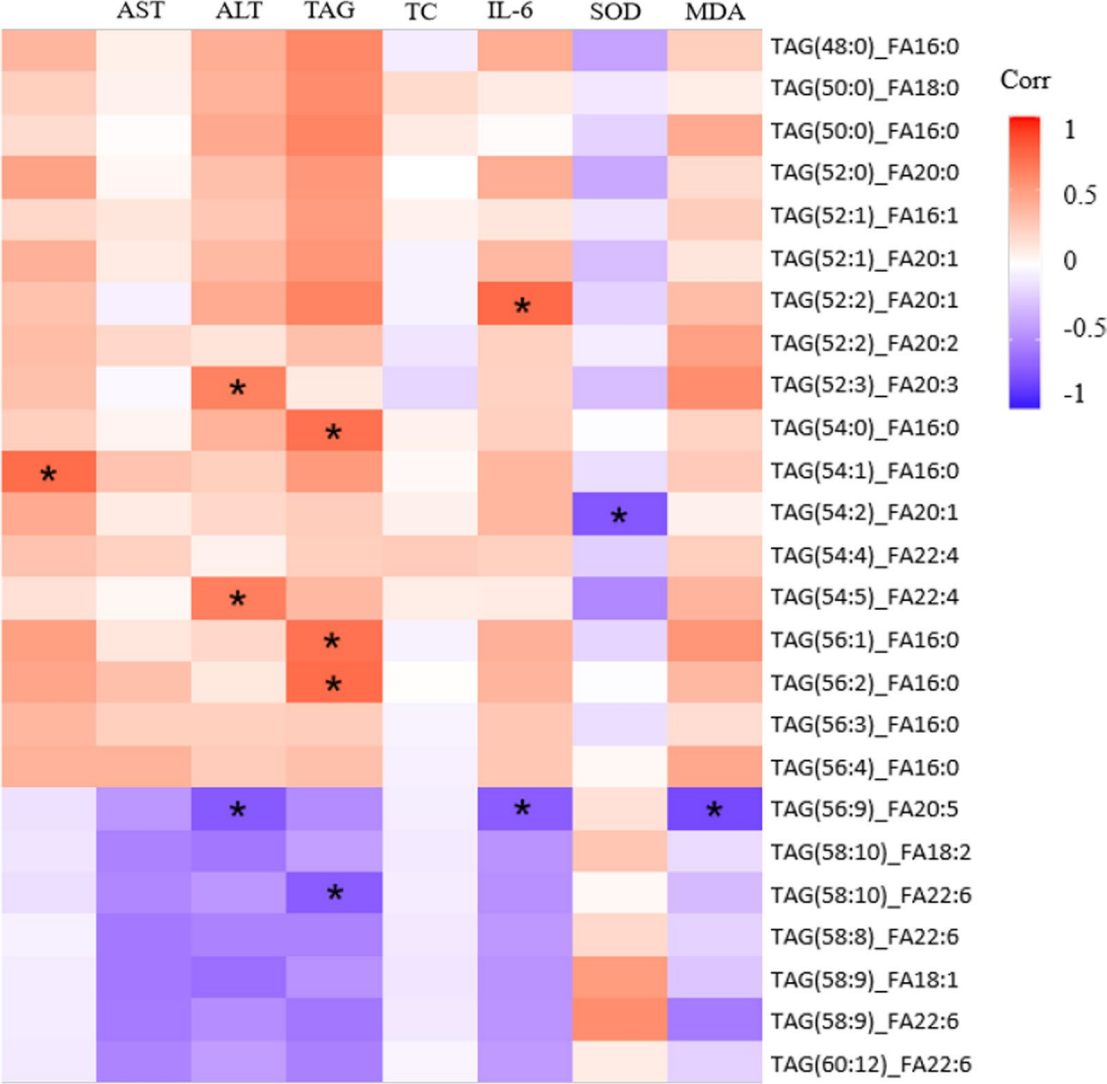
Correlation between the indicators of hepatic steatosis, lipid peroxidation and inflammation and the top 25 significantly changed triacylglycerolls (TAGs). Red represents positive correlation and blue represents negative correlation. *P < 0.05

In the analysis of the correlation between differential TAGs and the indicators of hepatic steatosis, lipid peroxidation and inflammation, we selected the top 25 changed TAGs, including 18 up-regulated TAGs and 7 down-regulated TAGs. The data showed that plasma *TNF-α* was positively correlated with TAG(54:1)_FA16:0. Plasma *IL-6* was positively correlated with TAG(52:2)_FA20:1 and inversely correlated with TAG(56:9)_FA20:5. ALT level in plasma was positively correlated with TAG(52:3)_FA20:3 and TAG(54:5)_FA22:4, while inversely correlated with TAG(56:9)_FA20:5. Total TAG level in liver was positively correlated with TAG(54:0)_FA16:0, TAG(56:1)_FA16:0 and TAG(56:2)_FA16:0, but inversely correlated with TAG(58:10)_FA22:6. MDA and SOD level was inversely correlated with TAG(56:9)_FA20:5 and TAG(54:2)_FA20:1, respectively (Fig. 5).

## Discussion

To the best of our knowledge, this is the first study to comprehensively investigate the lipids changes in response to long-term alcohol intake using the method of absolute quantitative lipidomics. We found that alcohol intake significantly aggravated the hepatic steatosis, liver injury, lipid peroxidation, as well as increased the inflammation level in mice liver. Lipidomics analyses revealed that alcohol intake obviously dysregulated the metabolism of ten lipids classes in liver, including TAGs, diacylglycerols, phosphatidylethanolamines, ceramides, phosphatidylcholines, sphingomyelins, free fatty acids, lysophosphatidylcholines, lysophosphatidylethanolamines and cholesterol esters. Among them, TAG accounts for the largest proportion of the altered lipids.

TAGs are the predominant lipids that accumulate in liver of ALD patients, with the form of lipid droplets [14]. Previous studies always focus on the mechanisms of the formation and growth of lipid droplets, in terms of the expression of genes and proteins involved in lipogenesis, lipid export and fatty acids oxidation, such as SREBP-1, PPARα, AMPK and CPT1 [15–20]. One interesting finding of the present study was that even though the total amount of TAGs was increased by alcohol exposure, some TAGs are still dramatically decreased. Long-term alcohol exposure mainly down-regulated the TAGs containing docosahexaenoic acid (C22:6n-3) and eicosapentaenoic acid (C20:5n-3), with more double bond and longer carbon chain length. Our result also found that the decreased TAG(56:9)_FA20:5 was inversely associated with ALT, MDA and *IL-6* levels, TAG(58:10)_FA22:6 was inversely associated with total TAG level, indicating that lack of these TAGs might be a potential risk factor for ALD development. However, the biological effects of these decreased TAGs on ALD pathogenesis are not clear at this stage. Thus further research on these TAGs are necessary.

Generally, n-3 polyunsaturated fatty acids have always shown protective effect on human health. Substantial results from both laboratory investigations and epidemiological studies suggested that lack of n-3 polyunsaturated fatty acids in diet might result in undesirable adverse outcomes [21–23]. Studies focus on the effect of n-3 polyunsaturated fatty acids diet on ALD have demonstrated that, both flaxseed oil (rich in C18:3n-3) and fish oil (rich in C20:5n-3 and C22:6n-3) diet exert protective effects against ALD [11, 24]. Surprisingly, our results showed the levels of free fatty acids of C20:5n-3 and C22:6n-3 in mice liver were significantly increased in response to alcohol exposure. In addition, another interesting phenomenon was that ten of the twelve changed diacylglycerols were up-regulated. As we all know, diglycerides and free fatty acids are formed by the breakdown of TAGs. Given the above-mentioned characters of the down-regulated TAGs that most of them contained n-3 polyunsaturated fatty acids, it is reasonable to speculate that the increased diacylglycerols and free fatty acids of C20:5n-3 and C22:6n-3 are resulted from the decomposition reaction of these TAGs. This is a novel message to us that chronic alcohol not only promoted the synthesis of TAGs, but also promoted the breakdown of some specific TAGs. Future research on these TAGs were warranted to exactly evaluate their physiological functions and their effects on ALD pathogenesis.

In the present study, we also found that the 3 changed LPCs and 3 LPEs in response to alcohol exposure were all down-regulated. Compared with the effects on TAGs and free fatty acids metabolism, the effect of alcohol intake on LPC and LPE in liver is less well established but may be increased [7, 25]. LPC and LPE always server as the precursors of lysophosphatidic acid (LPA). LPA is an endogenous bioactive lipid, and implicated in a variety of processes, such as lymphocyte homing [26], vascular homeostasis [27], demyelination and neuropathic pain [28], stem cell physiology [29], cellular homeostatic and immune [30]. Animal study indicated that LPA3 (receptor of LPA) deficiency led to abnormal of hepatocytes, and increased the susceptibility of mice to liver cancer [31]. These results indicated that the alcohol-induced perturbations in LPC and LPE level may contribute to the ALD pathogenesis by reducing LPA level.

Altered hexosylceramides (HCER) metabolism is an emerging area of ALD pathogenesis. Hexosylceramides is converted from ceramides in human cells [32]. Ceramides are a kind of bioactive lipids that can accumulate in the liver of both human and animal model of ALD, inducing oxidative stress, impairing insulin signaling, and increasing lipoprotein aggregation [33–35]. Alcohol exposure can modulate the de novo synthesis of ceramide, and mRNA of three ceramide synthases (CERS1, CERS5, and CERS6) are significantly increased in human livers with advanced ALD [34]. Besides, CERS6 is up-regulated in both experimental models of ALD and alcoholic steatosis patients, and can positively modulate the accumulation of lipid droplets [36]. Our data showed that 7 ceramides were up-regulated, while 6 HCERs were down-regulated, especially the HCer(18:1/22:0), with the top VIP value among all the down-regulated lipids. These results indicated that not only the up-regulated of ceramides, but also the down-regulated of hexosylceramides play a critical role in ALD pathogenesis. Elevating HCer(18:1/22:0) to a normal level might be a novel therapeutic opportunity for ALD management and warrants more further investigation.

## Conclusions

The present study demonstrated that alcohol intake dysregulated many aspects of hepatic lipid metabolism. TAG accounts for the largest proportion of the altered lipids. Even though the TAG amount were significantly increased in response to alcohol exposure, some specific TAGs containing docosahexaenoic acid (C22:6n-3) and eicosapentaenoic acid (C20:5n-3), with more double bond and longer carbon chain length were still dramatically decreased. In addition, chronic alcohol consumption significantly decreased several LPC, LPE and HCER levels, especially HCer(18:1/22:0), with the top VIP value among all the down-regulated lipids. These results might unveil novel risk factor for ALD, and could potentially spark interest among scientists to further investigate the important role of various lipids on ALD pathogenesis.

## Supplementary Information

**Additional file 1: Table S1.** The list of internal standards used in lipidomics analysis.

## Data Availability

The datasets used in the present study are available from the corresponding author on reasonable request.

## Abbreviations

ALD: Alcoholic liver disease
AF: Alcohol-fed group
PF: Pair-fed control group
AST: Aspartate aminotransferase
ALT: Alanine aminotransferase
TC: Total cholesterol
TAG: Triacylglycerol
H&E: Haematoxylin and Eosin
QC: Quality control
SD: Standard deviations
PCA: Principal component analysis
OPLS-DA: Orthogonal partial least squares discriminant analysis
VIP: Variable influence on the projection
LPC: Lysophosphatidylcholines
LPE: Lysophosphatidylethanolamines
LPA: Lysophosphatidic acid
HCER: Hexosylceramides
PE: Phosphatidylethanolamines
DAG: Diacylglycerols
PC: Phosphatidylcholines
CE: Cholesterol esters
CER: Ceramides
DCER: Dihydroceramides
SM: Sphingomyelins
FFA: Free fatty acids
