# Supplementary material for: Absolute quantitative lipidomics reveals lipids profiling in liver of mice with early-stage alcoholic liver disease
Source: Nutr Metab (Lond). 2022 Jul 5;19:42. doi: 10.1186/s12986-022-00679-z (PMC9254412; doi:10.1186/s12986-022-00679-z)
Supplement: Supplementary file 1 — Additional file 1: Table S1. The list of internal standards used in lipidomics analysis. [file 12986_2022_679_MOESM1_ESM.doc]

**Supply Table 1**. The list of internal standards used in lipidomics analysis.

|  | Abbreviation | Internal standard name |
| --- | --- | --- |
| Phosphatidylethanolamines | PE | PE(15:0/18:1) |
| Diacylglycerols | DAG | DAG(15:0/18:1) |
|  |  | DAG(16:0/22:6) |
|  |  | DAG(16:0/18:0) |
|  |  | DAG(16:0/18:1) |
|  |  | DAG(16:0/20:5) |
|  |  | DAG(16:0/18:2) |
|  |  | DAG(16:0/16:0) |
|  |  | DAG(16:0/18:3) |
|  |  | DAG(16:0/20:4) |
| Phosphatidylcholines | PC | PC(15:0/18:1) |
| Cholesterol esters | CE | CE(20:3) |
|  |  | CE(16:0) |
|  |  | CE(18:2) |
|  |  | CE(20:5) |
|  |  | CE(18:1) |
|  |  | CE(16:1) |
|  |  | CE(20:4) |
|  |  | CE(22:6) |
| Ceramides | CER | Cer(18:1/16:0) |
| Sphingomyelins | SM | SM(24:1) |
|  |  | SM(16:0) |
|  |  | SM(18:1) |
|  |  | SM(24:0) |
| Hexosylceramides | HCER | HexCer(18:1/16:0) |
| Lysophosphatidylcholines; | LPC | LPC(18:1) |
| Lysophosphatidylethanolamines | LPE | LPE(18:0) |
| Dihydroceramides | DCER | Cer(18:0/16:0) |
| Free fatty acids | FFA | FFA(16:0) |
|  |  | FFA(17:1) |
| Triacylglycerol | TAG | TAG(54:5/FA20:4) |
|  |  | TAG(52:1/FA18:0) |
|  |  | TAG(54:4/FA20:3) |
|  |  | TAG(52:4/FA18:3) |
|  |  | TAG(52:3/FA18:2) |
|  |  | TAG(50:1/FA16:0) |
|  |  | TAG(48:1/FA18:1) |
|  |  | TAG(56:7/FA22:6) |
|  |  | TAG(52:2/FA18:1) |
